# Synthesis and Characterization of Controlled Nitric Oxide Release from *S*-Nitroso-*N*-Acetyl-d-Penicillamine Covalently Linked to Polyvinyl Chloride (SNAP-PVC)

**DOI:** 10.3390/bioengineering5030072

**Published:** 2018-09-05

**Authors:** Sean P. Hopkins, Megan C. Frost

**Affiliations:** Department of Biomedical Engineering, Michigan Technological University, Houghton, MI 49931, USA; sphopkin@mtu.edu

**Keywords:** nitric oxide releasing polymer, controlled release, photoinitiated, PVC, biocompatibility

## Abstract

Polyvinyl chloride (PVC) is one of the most widely used polymers in medicine but has very poor biocompatibility when in contact with tissue or blood. To increase biocompatibility, controlled release of nitric oxide (NO) can be utilized to mitigate and reduce the inflammatory response. A synthetic route is described where PVC is aminated to a specified degree and then further modified by covalently linking *S*-nitroso-*N*-acetyl-d-penicillamine (SNAP) groups to the free primary amine sites to create a nitric oxide releasing polymer (SNAP-PVC). Controllable release of NO from SNAP-PVC is described using photoinitiation from light emitting diodes (LEDs). Ion-mediated NO release is also demonstrated as another pathway to provide a passive mechanism for NO delivery. The large range of NO fluxes obtained from the SNAP-PVC films indicate many potential uses in mediating unwanted inflammatory response in blood- and tissue-contacting devices and as a tool for delivering precise amounts of NO in vitro.

## 1. Introduction

Nitric oxide (NO) is a free radical, bioregulatory molecule in the body with a variety of important functions such as controlling vasodilation of blood vessels, acting as a neurotransmitter, and other important cellular signaling pathways which are dependent on the NO flux being generated [1,2,3]. Within the vasculature, endogenous *S*-nitrosothiols (RSNOs) such as *S*-nitrosoglutathione also help regulate these signaling processes along with generating NO at various locations within the body [4]. The production of NO is accomplished through specific enzymatic pathways based on different isoforms of nitric oxide synthase (NOs), depending on the specific physiological location in the body. When NO is released into the bloodstream by endothelial cells, it interacts with platelets, contributing to the non-adhesive properties of vasculature by controlling intracellular calcium regulation [5,6]. If NO production is impaired in the endothelium, the risk for atherosclerosis and hypertension drastically increases. Macrophages are another cell type that utilize the benefits of NO by promoting cytotoxic activity specifically towards viruses, bacteria, protozoa, and tumor cells [7]. 

The popularity of NO has led to a wide variety of materials capable of mimicking these beneficial properties. Incorporation of a widely-used RSNO, *S*-nitroso-*N*-acetyl-d-penicillamine (SNAP), into different polymer matrices has been accomplished by either direct physical blending, impregnation through swelling in SNAP solutions, or covalent attachment to the polymer [8,9,10]. Each method has its own benefits and drawbacks. Blending NO donors within a polymer matrix can give you precise control over exactly how much NO is released from a specific material by varying your donor concentration but has issues with potential leaching of the donor and by-products into the cellular environment which can be cytotoxic. Covalent attachment can prevent this issue but is more difficult to control in terms of total NO loading and release capacity. This has led to the development of a variety of polymers modified with SNAP to utilize the beneficial effects of NO.

Polymers such as chitosan have been developed with covalently bound SNAP and macromolecules such as dendrimers have also been synthesized with the same strategy [11,12]. The hemocompatibility of several blended and swelled SNAP-based polymers have been tested in extracorporeal circuits (ECCs) to further demonstrate how NO-releasing materials can mimic healthy vasculature [8,13,14]. Simultaneously, these polymers have been demonstrated to be effective antimicrobial agents against a broad range of gram positive and negative bacteria, which is another popular use for NO [15,16,17]. As antibiotic resistant bacteria continue to be an issue within the medical field, the development of more novel NO-releasing polymers has become more prevalent as a possible solution.

PVC is the most widely used polymeric material in medical applications including blood bags, heart lung bypass sets, and endotracheal tubes [18]. Around 25% of all biomedical devices contain some form of PVC due to its adjustable material properties and interactions with certain plasticizers and fillers [19]. PVC also has a very low cost to manufacture compared to other medical grade polymers and has a strong resistance to external chemical components. It is simple to chemically modify as the free chlorine reactive groups allow for the attachment of other chemical components to further enhance the surface chemistry of PVC. Previously, NO-releasing PVC was created by blending *N*-diazeniumdiolates within the polymer matrix and covalently binding it to a modified version of the polymer [20]. 

Herein, we describe the synthesis and characterization of a novel NO-releasing PVC based polymer (SNAP-PVC) and demonstrate its wide range of tunable NO-releasing properties which depend on the trigger mechanism. Primary amine groups are first attached to the PVC backbone, allowing SNAP groups to then modify the polymer to make it capable of NO release. Controlled release over a large range of NO fluxes was then demonstrated using light. The novel material described in this report imparts NO release from a covalently bound RSNO using a straightforward, cost-effective means to produce a robust, tunable NO releasing material. In contrast to *N*-diazeniumdiolates, NO release from RSNOs can be triggered through multiple different mechanisms, allowing SNAP-PVC to have a wide array of potential applications [21]. By using an RNSO functionalized polymer, SNAP-PVC is able to have an active tunable control of NO release for a variety of situations along with the possibility to create a passive release system.

## 2. Materials and Methods 

### 2.1. Materials

Polyvinyl chloride (average M_w_ = 233,000, M_n_ = 99,000) (PVC), ethylenediamine, 5,5’-dithiobis (2-nitrobenzoic acid), triethylamine, 1,4,8,11-tetraazacyclotetradecane (cyclam), *N*-acetyl-d-penicillamine (Fluka), and concentrated hydrochloric acid were obtained from Sigma-Aldrich (St. Louis, MO, USA). ATTO-TAG^TM^ FQ Amine-Derivatization Kit was purchased from Invitrogen (Grand Island, NY, USA). *Tert*-butyl nitrite (90% technical grade, Acros Organics) was purchased from Fisher Scientific. Magnesium sulfate (MgSO_4_), copper (II) bromide (CuBr_2_), L-ascorbic acid sodium salt, and acetic anhydride (Alfa Aesar) were purchased from VWR (West Chester, PA, USA).

### 2.2. Synthesis of NAP-Thiolactone

The procedure reported by Moynihan and Robert was followed to synthesize a self-protected *N*-acetyl-d-penicillamine (NAP) thiolactone [22]. Briefly, 5 g of *N*-acetyl-d-penicillamine was dissolved in 10 mL of pyridine while 10 mL of acetic anhydride and 10 mL of pyridine were mixed in a separate container. Then, both solutions were cooled in an ice bath for 1 h before being combined. The mixture was stirred at room temperature for approximately 24 h until the solution turned light red. The solution was rotary-evaporated at 45 °C to remove pyridine. The temperature was then increased to 60 °C until most of the remaining solvent was removed to obtain an orange viscous liquid. The resulting product was then dissolved in 20 mL of chloroform and washed and extracted three times with 20 mL of 1 M HCl. MgSO_4_ was added to the chloroform solution to remove any water and was then removed by vacuum filtration. The chloroform was removed by rotary evaporation at room temperature and the resultant crystals were collected and rinsed with hexanes and filtered. Light yellow/white colored crystals were recovered and vacuum dried overnight.

### 2.3. Synthesis of SNAP-PVC

**Figure 1:**
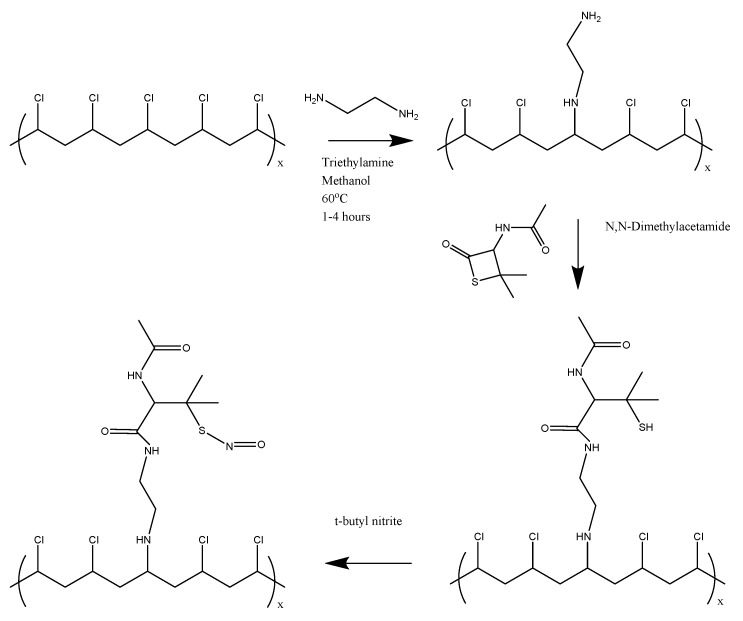
Synthesis route of the nitric oxide-releasing polymer (SNAP-PVC).

Figure 1 shows the overall reaction scheme to synthesize SNAP-PVC. Aminated PVC was first synthesized using a modified procedure from Tinkilic et al. [23]. Briefly, 2.5 g of PVC was suspended in 50 mL of methanol and 11 mL of triethylamine and heated to 60 °C. 15.25 mL of ethylenediamine was then slowly added to the heated solution. The reaction mixture was allowed to reflux for a designated amount of time at 60 °C. The resulting light yellow polymer powder was then filtered and washed thoroughly with water, methanol, 1 M HCl, and water, in that order, before being dried under vacuum. 

For NAP attachment, 200 mg of PVC-NH_2_ was first dissolved in 10 mL of anhydrous *N*,*N*-dimethylacetamide (DMAC). 60 mg of NAP-thiolactone was then added to the mixture and stirred overnight. 2 mL of the NAP-PVC solution was taken out and nitrosated using 0.5 mL of *t*-butyl nitrite. The *t*-butyl nitrite was first treated to chelate any copper stabilizer using a 30 mM aqueous cyclam solution (1,4,8,11-tetraazacyclotetradecane). For the chelation step, approximately 3 mL of *t*-butyl nitrite and 5 mL of 30 mM cyclam were mixed and stirred vigorously. The cyclam was then removed and the *t*-butyl nitrite was rewashed with more cyclam a total of three times before the cleaned *t*-butyl nitrite was extracted and stored at 2 °C. The light yellow/clear solution of PVC-thiolactone turned a light green-yellow color after being allowed to react with *t*-butyl nitrite for 30 min. The SNAP-PVC solution was then cast into polytetrafluoroethylene (PTFE) rings and left to dry under vacuum overnight to form a thin polymer film. A plasticizer additive could be dissolved into the solution before casting the film to give more elastic properties as pure PVC can be somewhat brittle. Non-plasticized PVC was used for the experiments being described.

### 2.4. Polymer Characterization

The quantification of the degree of amination of the PVC was accomplished using the ATTO-TAG FQ test for primary amines. Fluorescence measurements were completed using a 96-well plate reader (BioTek Instruments, Winooski, VT, USA) and a glycine standard was used to form a calibration curve. The fluorescent tag was maximally excited at 450 nm with an emission maximum at 550 nm [24]. Once the aminated PVC reacted with NAP-thiolactone, the primary amine disappeared and a free thiol was exposed on the polymer backbone. Ellman’s test for thiols was done to quantify the amount of thiols grafted onto the polymer using 5,5’-dithiobis (2-nitrobenzoic acid) (DNTB), following a modified protocol developed by George Ellman [25]. Absorbance was then taken at 412 nm after the reaction with DTNB persisted for 1 h using a 96-well plate reader. FTIR was used to monitor the reaction progress by following the important polymer functional groups (Mattson Genesis II). CHN combustion analysis was performed on a Costech 4010 Elemental Combustion System to quantify the overall nitrogen content in the various aminated PVC iterations.

### 2.5. Nitric Oxide Release by Photoinitiation

The light source used for photoinitiated NO release from SNAP-PVC films was 460 nm blue VAOL-5GSBY4 LED obtained from Mouser Electronics Inc. (Mansfield, TX, USA). A 130 Ω resistor was connected in series with the LED and a variable voltage source was applied to the system. The emission spectra and relative intensity of the LED with respect to drive current was shown to be linear over the currents being used to power the LED for the study and was characterized by Starrett et al. [24].

### 2.6. Nitric Oxide Measurements

Nitric oxide release from the polymer was directly measured by the chemiluminescent reaction of NO with ozone using a Sievers 280i Nitric Oxide Analyzer (Zysense, LLC, Frederick, CO, USA). SNAP-PVC films with a diameter of 5.5 mm were placed inside an amber glass sample holder with an inlet nitrogen sweep gas flowing at 200 mL min^−1^. The LED was mounted in the top of the sample holder, 5 cm above the film to be tested. Varying voltage levels were used (0, 3, 4.5, 6, 7.5 volts) at 130 Ω to demonstrate the control of the NO flux from the polymer film. Total NO release of the polymer films was done by cleaving the sulfur-nitroso bond through copper-mediated decomposition following a procedure developed by Frost et al. [26]. A solution of 10 mM CuBr_2_ and a 100 mM solution of L-ascorbic acid sodium salt was used for the NO quantification. 2 mL of the CuBr_2_ solution were placed in a glass sample holder containing a weighed SNAP-PVC film. After 5 min, 500 µL of the 100 mM L-ascorbic acid solution was then added to reduce the Cu^2+^ ions to Cu^+^, enhancing the sulfur-nitroso bond cleaving. The test was continued until all of the NO reservoir was depleted.

## 3. Results

### 3.1. Synthesis of Covalent Bound SNAP to PVC

Several strategies were implemented to optimize the amount of NO release from the SNAP-PVC materials. The overall goal was to covalently attach SNAP in the highest capacity, while still being able to maintain precise control of NO release via photoinitiation. The initial amination step with ethylenediamine was varied by reaction time for 1, 2, and 4 h. This was done to optimize the greatest amount of reactive primary amine groups on the PVC resin backbone. When reacted for too long, primary amine end groups react and crosslink with separate PVC polymer chains, eliminating the possibility for SNAP attachment and greatly reducing the solubility of the PVC. On the other hand, too short of a reaction time will limit the available primary amine sites, thereby reducing the overall amount of SNAP attached to the polymer backbone. Once completely synthesized, it was found that the 2 h ethylenediamine reaction time SNAP-PVC was optimal for maximal NO loading and was used for all full testing and characterization.

### 3.2. FTIR Analysis

**Figure 2:**
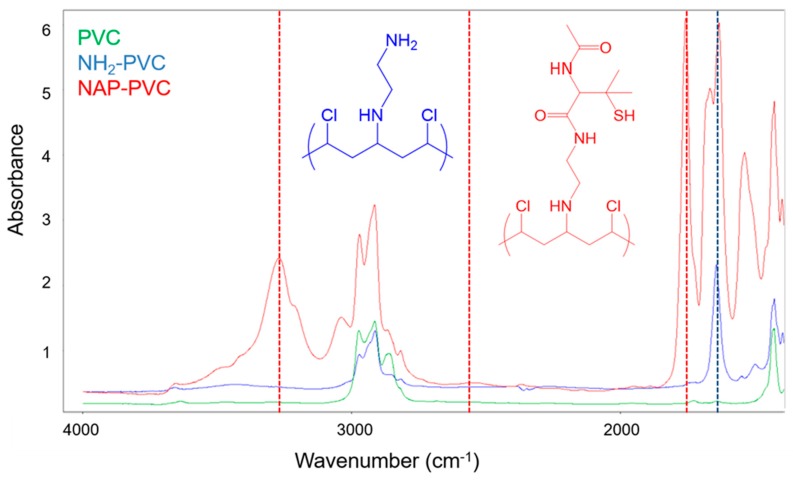
FTIR of Polyvinyl chloride (PVC) (green), PVC-NH_2_ (Blue), and *N*-acetyl-d-penicillamine-PVC (NAP-PVC) (Red).

Each step of the reaction schematic shown in the Figure 1 for the SNAP-PVC synthetic route was verified using FTIR (Figure 2). After attaching ethylenediamine to the PVC backbone, primary amines and secondary amines were present in the spectrum which was seen in the N-H bending at 1641 cm^−1^. The attachment of NAP-thiolactone to the free primary amine sites indicated the formation of NAP-PVC as the presence of a carbonyl peak is evident at 1750 cm^−1^ along with a broad, short thiol S-H peak at around 2550 cm^−1^ and an amide N-H stretch peak at 3270 cm^−1^. Additionally, the secondary amine peaks also increased with NAP-PVC because one primary amine was converted to a secondary amine and there was an additional secondary amine present in the newly attached acetylpenicillamine group.

### 3.3. Quantification of Functional Groups

Quantification of the amination of PVC was verified through doing the ATTO-TAG FQ test for primary amines. Once the NAP-thiolactone ring opened and reacted with the primary amine sites on the polymer backbone, free thiol groups were exposed. To identify the extent to which this reaction was occurring, Ellman’s test for thiols was done. The quantification of primary amine and thiol functional groups were normalized per milligram of polymer. Primary amine quantification showed 0.201 ± 0.013 µmol/mg of PVC-NH_2_. Thiol quantification showed 0.153 ± 0.009 µmol/mg of NAP-PVC. This was a 76.1% conversion rate of primary amine to react with the thiolactone. The resultant exposed free thiol groups where then nitrosated to form the SNAP group. To verify the total NO loading capacity, copper mediated NO release was performed on the nitrosated polymer (SNAP-PVC) and showed the NO released to be 0.0392 ± 0.0039 µmol per milligram of polymer, which is a total of 25.6% NO loaded for the potential thiol groups present.

### 3.4. NO Release from SNAP-PVC

The physiological NO flux for healthy vasculature is estimated to be in the range of 0.5–4 × 10^−10^ mol·cm^−2^·min^−1^, therefore this is the target surface flux goal from NO-releasing materials. The specific NO flux generated varies depending on the physiological status of the patient and the location in the body. Locations in the vasculature with higher sheer stress will promote higher local NO release from vascular endothelial cells [27]. After damaging the vascular walls from an implanted device, more NO release may be initially required to prevent unwanted thrombosis and inflammation, potentially leading to and neointimal hyperplasia in the affected area [28]. Having a material that is able to adjust the NO flux increases the number of applications a hydrophobic polymer coating could have in a medical setting.

**Figure 3:**
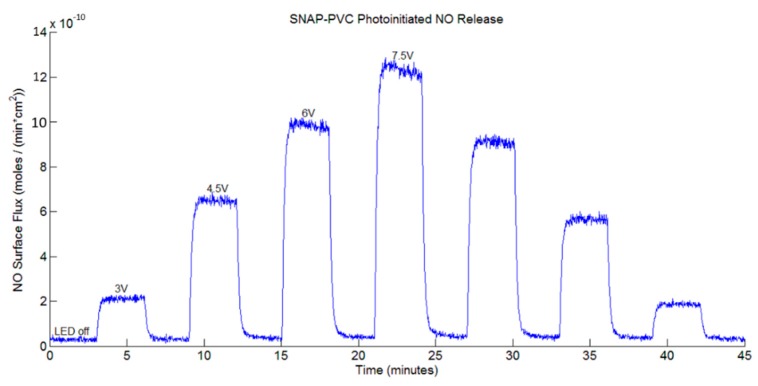
Representative nitric oxide (NO) release profile of a SNAP-PVC film (5.5 mm dia., 0.1 mm thick disc) from photoinitiated NO release using a 460 nm LED with varied applied voltages, resulting in varied levels of light energy impinging upon the film while the resistance was held at 130 Ω.

Figure 3 shows the NO release profile from SNAP-PVC films (diameter 5.5 mm, thickness 0.1 mm) at varying applied voltage levels using a 460 nm wavelength LED with no bathing solution present (i.e., dry films exposed to light). Nitrogen sweep gas carried the NO released by photoinitiation to the chemiluminescence detector to result in a stair-step profile based on the voltage supplied to the LED. The profile showed the wide range of control in NO flux that the polymer was able to obtain (~0–12 × 10^−10^ mol·cm^−2^·min^−1^). As the light emitted from the LED was adjusted (by varying the range of applied potential between 0–7.5 V), there was little decay in the NO being released at each level, showing the stability of the NO reservoir within the polymer. Being able to control the NO flux of a material can be a useful tool for in vitro experiments. Since cells in the body interact with NO at specific flux ranges of periods of time, directly adding a soluble NO donor such as SNAP would not accurately mimic what is seen in vivo. These donors continually decompose over time in solution, resulting in unpredictable burst releases of NO that are difficult to predict [29]. Cell culture experiments would benefit from a material that can deliver precise fluxes of NO over time as certain therapeutic administrations of NO may be necessary to achieve the desired end result.

**Figure 4:**
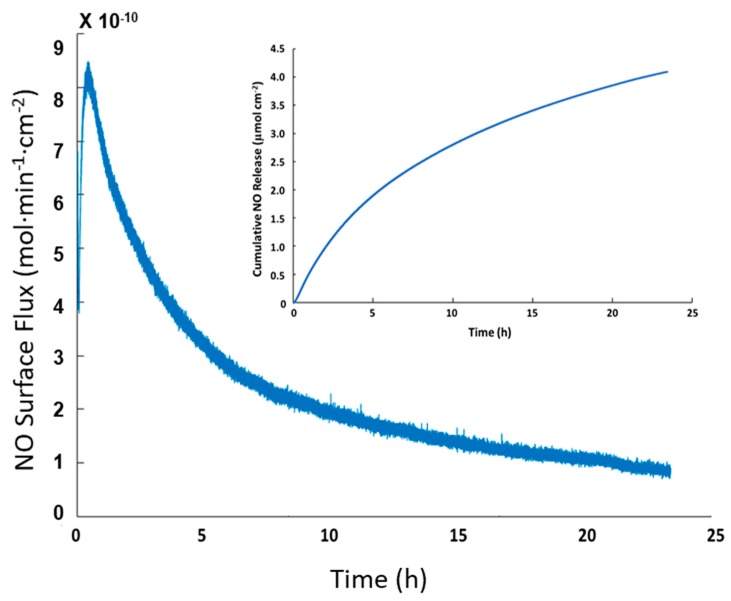
Passive NO release profile of a representative SNAP-PVC film (5.5 mm dia., 0.1 mm thick disc) placed in PBS at 37 °C for 24 h (inset shows the cumulative NO release from the film). The NO release was initiated by trace levels of transition metal ions present in PBS.

NO release of SNAP-PVC in phosphate buffered saline (PBS) at 37 °C was also tested to show the release at physiological conditions. Trace transition metal ions are able to penetrate the polymer film and interact with the reactive SNAP groups (PVC is a popular polymer used in ion selective electrodes) [30]. Most hydrophobic polymers tend to resist ion diffusion, which is seen with medical grade silicone rubbers that are popular for in vivo applications [31]. The ability of transition metal ions and ascorbate to diffuse into PVC provides an additional pathway to stimulate the NO response for SNAP-PVC, as light mediated NO release will not always be possible. In some scenarios, having a level of passive release over time may be the optimal route for NO generation. This follows a similar release profile as seen with N-diazeniumdiolates NO donors, but is able to release lower amounts over a longer period of time. The passive release of SNAP-PVC in PBS at 37 °C is shown in Figure 4. The amount of NO generated in this manner could be controlled by adjusting the diffusion of ions into the PVC by applying certain polymer topcoats and/or varying the amount of SNAP-PVC present by controlling the thickness of the SNAP-PVC films or blending SNAP-PVC with standard PVC.

**Figure 5:**
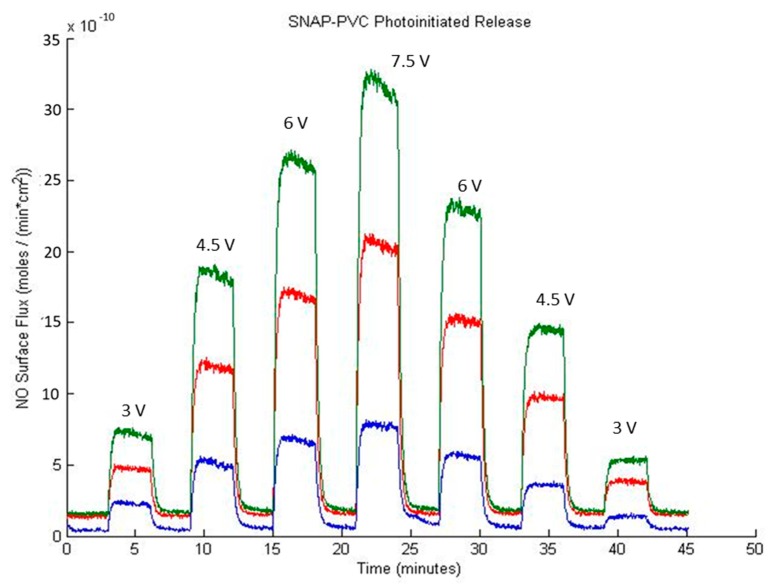
SNAP-PVC photoinitiated NO release at different synthetic reaction times with ethylenediamine at 37 °C in nitrogen where 2 h (green) demonstrated a higher NO release potential than 1 h (red) and 4 h (blue). NO release from a SNAP-PVC film (5.5 mm dia., 0.1 mm thick) was initiated using a 460 nm LED with a 130 Ω resistor and varying applied potentials as noted on the face of the graph.

The NO capacity and release demonstrated by SNAP-PVC could potentially be much larger as there were many unreacted chlorine sites on the backbone of the polymer. A longer reaction time with ethylenediamine could be implemented to achieve a higher degree of amination but complications with crosslinking and solubility occur as PVC becomes more dechlorinated [32]. This limits the NO storage capacity of SNAP-PVC through using a component like ethylenediamine as an aminating agent. PVC also becomes darker in color the more dechlorinated it becomes, which would hinder the NO release by photoinitiation as light penetration through the film would be limited. As mentioned previously, NO release from films reacting with ethylenediamine for 1, 2, and 4 h were tested, and 2 h was found to have the highest capacity of NO while still allowing photolytic cleavage of the RSNO functional group. A separate LED study varying the level of light impinging on the polymer films was performed on all three types of SNAP-PVC films while at 37 °C and is shown in Figure 5.

**Figure 6:**
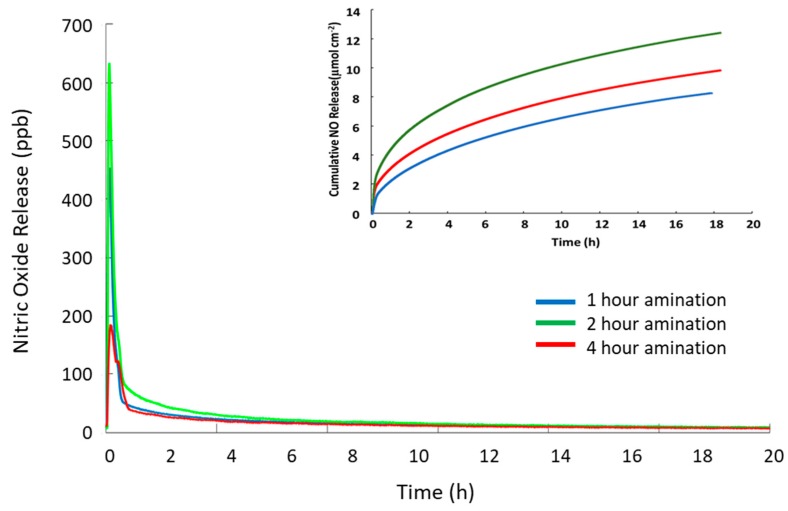
Total nitric oxide release from SNAP-PVC polymer synthesized with different reaction times for initial amination. Chemiluminescence detection was used to record total NO release triggered with UV light. The SNAP-PVC were reacted with ethylenediamine for 1 h (blue), 2 h (green), and 4 h (red). The measurements were made with cast films of SNAP-PVC that were allowed to cure under ambient conditions protected from light.

In order to optimize the amination reaction time, PVC polymers were reacted for 1, 2 and 4 h and then converted to SNAP-PVVC by reaction with the thiolactone, followed by nitrosation. The polymers were cast into films and irradiated with high intensity UV light to force complete release of NO. The total NO release from the resultant polymers was recorded with chemiluminescence detection. Figure 6 shows that the 1 h reaction time SNAP-PVC (blue trace) released the lowest levels of NO while the 2 h reaction time SNAP-PVC (green trace) released the highest level of NO for the same illumination. The 4 h amination time (red trace) released a greater total load of NO than the 1 h, but was significantly less total NO than the 2 h animation time. An explanation for this could be that the amination crosslinking at 4 h reaction time excessively interferes with the ultimate formation of the SNAP groups on the PVC backbone. This was further confirmed by CNH analysis that showed the increase in ethylenediamine reaction time corresponds to an increase in the nitrogen content but a decrease in primary amines at the 4 h reaction time (see Table 1), where the 4 h reaction time has 1/10 the primary amines present (0.018 µmol/mg of polymer compared to 0.201 µmol/mg of polymer for the 2 h reaction time) even though it has a high total nitrogen content by mass (0.20% N for 4 h reaction time compared to 0.17% N for 2 h reaction time). A higher level of amination with primary amines could possibly be achieved, thereby increasing the capacity to form SNAP by using a protected aminating agent instead of an accessible difunctional aminating agent such as ethylenediamine. This would allow the attachment of the reactive molecule to the backbone of the PVC chains with no crosslinking. The polymer could then be deprotected to expose the reactive group that could then be modified with SNAP. 

## 4. Conclusions

The simple, robust synthesis of NO-releasing SNAP-PVC that contained covalently bound SNAP functional groups was fully characterized. Optimization was completed to identify what degree of amination to the chlorine backbone of PVC promotes the most SNAP attachment. Using a 460 nm LED, controlled photoinitiated NO release was achieved. The material demonstrated its ability to cover a wide range of NO fluxes (0–35 × 10^−10^ mol·cm^−2^· min^−1^) that could be useful for potential in vitro studies that require c = varied levels of controlled NO delivery. This could greatly improve the potential applications for blood- and tissue-contacting PVC-based materials that are already used in medical settings. The material could also be used as implantable device coatings to improve biocompatibility or to prevent neointimal hyperplasia of vessels from its passive NO release capabilities. Future studies are being done to extend the life of NO-releasing polymers.

## Figures and Tables

**Table 1:** Primary amine content using the ATTO-TAG FQ Amine-Derivitization test and nitrogen content via CHN analysis of PVC aminated for 1, 2 and 4 h to observe the primary amine and nitrogen content as reaction time with ethylenediamine increases.

Sample	Primary Amine Content (µ mol/mg)	% N (by Mass)
PVC 1 h reaction time	0.036 ± 0.016	0.15
PVC 2 h reaction time	0.20 ± 0.013	0.167
PVC 4 h reaction time	0.019 ± 0.0023	0.20
